# Aggregating Twitter Text through Generalized Linear Regression Models for Tweet Popularity Prediction and Automatic Topic Classification

**DOI:** 10.3390/ejihpe11040109

**Published:** 2021-11-26

**Authors:** Chen Mo, Jingjing Yin, Isaac Chun-Hai Fung, Zion Tsz Ho Tse

**Affiliations:** 1 Department of Biostatistics, Epidemiology and Environmental Health Sciences, Jiann-Ping Hsu College Public Health, Georgia Southern University, Statesboro, GA 30458, USA; cm06957@georgiasouthern.edu (C.M.); cfung@georgiasouthern.edu (I.C.-H.F.); 2 Department of Electronic Engineering, The University of York, Heslington, York YO10 5DD, UK; zion.tse@york.ac.uk

**Keywords:** regression, social network, text data, document term matrix, odds ratio, relative risk, hurdle model

## Abstract

Social media platforms have become accessible resources for health data analysis. However, the advanced computational techniques involved in big data text mining and analysis are challenging for public health data analysts to apply. This study proposes and explores the feasibility of a novel yet straightforward method by regressing the outcome of interest on the aggregated influence scores for association and/or classification analyses based on generalized linear models. The method reduces the document term matrix by transforming text data into a continuous summary score, thereby reducing the data dimension substantially and easing the data sparsity issue of the term matrix. To illustrate the proposed method in detailed steps, we used three Twitter datasets on various topics: autism spectrum disorder, influenza, and violence against women. We found that our results were generally consistent with the critical factors associated with the specific public health topic in the existing literature. The proposed method could also classify tweets into different topic groups appropriately with consistent performance compared with existing text mining methods for automatic classification based on tweet contents.

## 1. Introduction

The use of social media has increased globally, and studies of social media have emerged in various areas, including public health research. Analysis of a wide range of health topics has been conducted using text data collected from different social platforms, like Facebook and Twitter [1,2,3]. However, many studies have revealed that one of the limitations of using data from social media for health-related research is the poor reliability and validity of the data [4,5]. The data observed in real-world social media usually show diverse combinations of terms in different sentences, producing a sparse document term matrix (DTM). Thus, there is a paucity of relevant data that can be used in data analysis. The problem of the diversity of terms in big text data leads to questions regarding the reliability and validity of information gleaned from social media for health-related studies [5].

The limitations of data reliability and validity have motivated us to develop an alternative method to provide statistical evidence for choosing keywords in public health studies. Due to the high dimensionality and great sparsity of text data, data scientists usually apply some low-rank approximation to reduce the number of keywords and thus shrink the dimension of a dataset [6,7]. However, the advanced computational techniques behind these methods and the interpretation of the results in ordinary language are very challenging for public health practitioners. When a dataset contains hundreds of terms, it is difficult to give logical explanations of the effect of individual terms, as well as the overall effect of all terms included in a text message. At the same time, many studies have only assessed the effect of single terms but not the overall influence of multiple terms [3,8]. Therefore, using text data on autism spectrum disorder (ASD) extracted from Twitter as an example, we propose to apply generalized linear regression models (GLMs) to study the effect of the wording of the text and the classification among different health topics. We choose to analyze the Twitter data for illustration of the proposed method, as it is a widely used platform for information exchange and has been used as a data-mining source to assess the population affected by health issues in many studies. For example, using text data extracted from Twitter, Beykikhoshk et al. [1] learned about the community affected by ASD, including their behaviors, concerns, and needs; Hswen et al. [3] studied the psychological characteristics of self-identified persons with ASD.

Particularly, Beykikhoshk et al. [1] provided the first study to use ASD-related Twitter data. They investigated and compared the performance of an automatic classification process based on three representations of terms in the tweet: (1) binary bag of words—either present in the corresponding tweet (=1) or not (=0); (2) integer bag of words (term count)—number of occurrences of that term in the corresponding tweet; and (3) tf-idf (term frequency-inverse document frequency)—normalized frequency of each term weighted by the inverse number of tweets containing the term. These methods can provide substantial accuracy in the classification process, but these text mining approaches are seldomly used by public health practitioners. Therefore, we proposed a rather straightforward approach to handle text data by applying regression analysis techniques, which is a more common approach and understood by most public health data analysts. Hence the primary purpose of this study is to give the first introduction or tutorial to public health practitioners to analyze text data based on regression analysis through two case studies.

In our study, the proposed method is applied to two cases: (1) to select influential key terms from tweets that are associated with more retweets for ASD-related tweets; and (2) to classify tweets between ASD and non-ASD topics based on keywords selected from tweets. The purpose of the first application is to investigate which term(s) will significantly impact the influence or popularity of a tweet, meaning it will result in a greater number of retweets. The primary outcome is the count of retweets; therefore, we use regression models for count data, such as Poisson, Negative-Binomial, and Hurdle models. To determine the key terms, we first estimate the relative risk (*RR*) for each term abstracted from the tweets of interest regarding the tweets’ popularity (i.e., the counts of retweets) by fitting a univariate count regression model for each term. The *RR* serves as the index for the choice of terms, where a term with a higher *RR* is more likely to be retweeted. The second application aims to find out whether a tweet belongs to the topic of interest according to the frequency of a selected list of keywords from the tweet. Our goal is to identify keywords through matching and counting of the frequencies of the words used in a health topic. The primary outcome for classification can be binary (indicating relevance of certain topic or not) or a categorical variable with more than two levels (indicating different topics). In our research, for illustration purposes, we focus on a binary model, with tweets being classified as either relevant to the topic or not. To determine the binary outcome, we use logistic regression models. The method can be easily extended to the situation of multi-category (≥2) classification using multinomial logistic regression models. First, we estimate the odds ratio (*OR*) of each key term that is shared for both groups, such as the ASD group (=1) and the non-ASD group (=0). The *OR* is the indicator of the association of terms with the topic of interest. The terms with higher *OR*s are more likely to appear in the topic.

Overall, the indicators, either *RR* or *OR*, help health practitioners learn about feedback from the general population online and further improve methods of communication. The proper choice of terms and the appropriate frequency of usage when delivering a health message help to attract the public’s attention and improve the implementation of a health strategy. Likewise, this method can be applied to electronic medical records (EMR) data for studying the text content in medical records [9].

In summary, the objectives of the study are to explore the feasibility of applying generalized linear models to summarize text data using the proposed AIS scores, which can be potentially useful for classification of topic-specific tweets, as well as to evaluate the impact and popularity of a topic-related tweet according to its wording (based on the aggregated scores of the associated terms). The proposed methods aim to ease the difficulty of the identification process compared with traditional NLP methods in order to provide straightforward interpretations.

## 2. Methods

### 2.1. Data Collection and Cleansing

Twitter data were retrieved from the Twitter Search Application Programming Interface (API) during the period from 1 to 30 April 2016, since April is autism awareness month. All tweets in English that included the keywords “Autism” or “ASD” (case-insensitive and including hashtags) were pulled from these dates (N = 553,154). To illustrate the proposed method, a 10% stratified random sample by date was then selected (n = 55,315).

The data were cleaned and analyzed using R 3.5.2 [10]. In this study, the terms extracted from variable “Text” served as predictors in the initial regression analysis. To eliminate ambiguous results, we removed some terms that appeared in all tweets for a certain topic; for example, “autism” and “ASD” were used to extract tweets on the topic of ASD, and they appeared in all ASD-related tweets. In addition, some terms and symbols, including punctuation marks, URL, @mention, numbers, hashtags, or spaces, were removed. Details of data cleaning are explained in the following steps:

The R function stringr::str_replace_all() [11] was used to remove topic words. For example, by the search query, every tweet in the ASD dataset contained “autism” and “asd”, so these words did not add value to the count model fitted using the ASD dataset only. In addition, for the classification model, these words had a dominant association with ASD and thus may have yielded too optimistic a performance. In order to obtain a fair and close-to-reality model evaluation, we removed “autism” and “asd”.

The URL, @mention, and words that were not numbers, letters, hashtag signs “#”, and spaces in the text were also removed, as well as the “RT” from a retweet.

Uppercase text was converted to lowercase using the R function tolower().

Moreover, the terms in the hashtags were usually shortened phrases, such as “azan”, “bercakap”, and “susah”, which are difficult to interpret. We compared results from models with and without hashtag words and found that the model without hashtag words offered better model fit as well as more meaningful interpretations. Therefore, we excluded hashtag terms in later analysis.

### 2.2. Data Preparation

After data cleaning, we applied the R package tm to create a DTM from the corpus. A DTM is a matrix with documents as rows and terms as columns. The transposition of a DTM is a term-document matrix (TDM). The tm package processes both DTM and TDM as sparse matrices [12].

The DTM was further cleaned by removing sparse terms that appeared in less than or equal to *k* (a pre-specified number, usually much less than *n*) documents. Sparse terms are usually noise, considered to be a limited source that has little contribution to the data analysis, and it was time-consuming to include all of these in the analysis. In our study, we conducted analyses using datasets with *k* equals 3 and 500 separately. Since both cases gave similar results, then *k* = 500 was more efficient as well as robust enough to use throughout the study. In summary, the steps for preparing the DTM for regression analysis are as follows:

1. The “Text” column in the dataset was read as a vector, and then a collection of documents was created into a Corpus object by the R function tm::Corpus(VectorSource()) [10]. As mentioned above, a document corresponds to a tweet in the dataset.
2. Punctuation was also removed using function tm::tm_map(x, removePunctuation), where x represented each tweet.
3. Then, tm::DocumentTermMatrix() was used to create a DTM, which contains all the terms that were listed in all texts and the frequency with which a term appeared in a document.
4. Sometimes, the dataset needed further cleaning because some off-interest terms were not obliterated. For example, in our study, URLs were not deleted and remained as terms starting with “HTTP”. Such terms were removed from the dataset.
5. Some terms were sparse, meaning they only appeared in a small number of tweets, and they had insufficient contribution to the potential association. Hence, those terms were removed from DTM based on the number of documents that included them.
6. The DTM, which contained the frequency of terms in each document, was merged with the outcomes of interest from the original dataset for future analysis.

### 2.3. The Proposed Data Aggregation Procedueres by Regression Models

**Figure 1:**
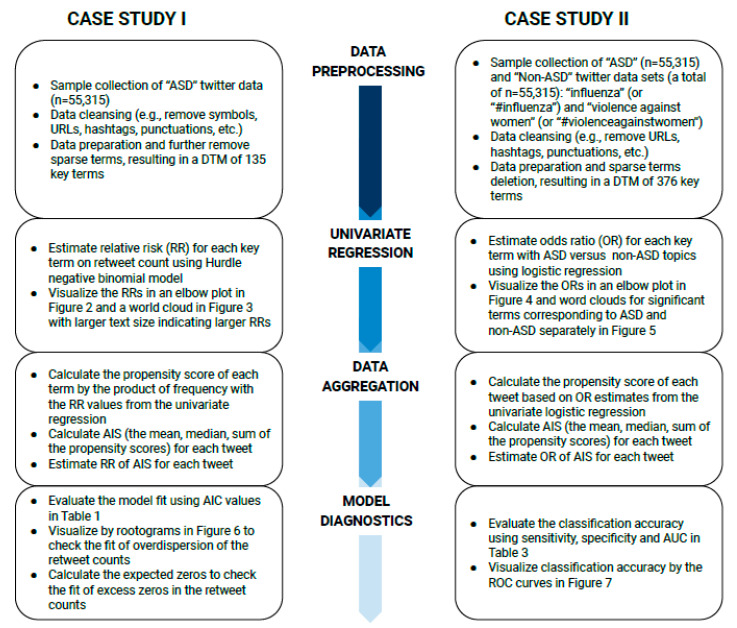
The method flowcharts for Case Studies I and II.

Even though some sparse terms were removed from the dataset, the reduced DTM still included many terms that would complicate the comprehension of the results. Therefore, we propose a new method that uses generalized linear regression models and standardizes the results across all terms by creating the aggregated influence scores (*AIS*s). The *AIS* measures the overall propensity of the key terms from each tweet to the topic of interest. The regression method measures the effect of terms, represented by either *RR* (for association) and *OR* (for classification), on respective outcomes. The *AIS* summarizes the effects from all terms to a single index by weights estimated from the regression model such that the number of predictors is greatly reduced. In this section, we use two case studies to illustrate the proposed application of the regression models for (1) association analysis in case study I and (2) classification analysis in case study II. Figure 1 summarizes the flowcharts of methods by each subsection for case I and case II.

#### 2.3.1. Case Study I: Influential Keyword Selection by Hurdle Negative-Binomial Model Using Retweet Count as the Outcome

In this study, the outcome of interest was the number of retweets. It was a count variable and represented how popular a tweet was. We hypothesized that the usage of certain terms could impact the popularity of a tweet, and we chose the predictors to be the frequencies of certain key terms in a tweet. The Poisson model is usually applied in count data analysis; however, it assumes that the mean and variance of the outcome are the same, which is not the case for Twitter data [13]. Later in the results section, for illustration and comparison purposes, we present Poisson models with log-linear and log-log link functions and conclude neither of the link functions work for Twitter data. Instead, the Negative-Binomial model was applied in the analysis due to the over-dispersed outcome in the Twitter data example (i.e., the variance of the outcome was markedly larger than its mean). In addition, the Twitter data had many zeros, and Hurdle models can be used to deal with the problem of excess zeros. The Hurdle model has two parts: a logistic model for zero counts (an outcome takes a value of one if a positive count or a value of zero if a zero count) and a zero-truncated count model (e.g., truncated Negative-Binomial model) for positive counts. Thus, for social media data, the analysis was also conducted with the Hurdle model with a zero-truncated Negative-Binomial model (hereafter referred to as the Hurdle model) for the nonzero case as it was more appropriate for the nature of the outcome data. We include both the regular and the Hurdle Negative-Binomial models for comparison purposes.

After data cleaning, the univariate analysis using the Hurdle Negative-Binomial model was performed for the 135 key terms to study the influence of the terms on the popularity of a tweet, followed by *AIS* calculation and evaluation of overall influence on the popularity of tweets. The detailed steps are presented below:

1. The *RR* of each term was calculated with the univariate Hurdle model:
  (1) $$\begin{array}{l} f_{\mathrm{hurdle}}(y_{i};x_{i},z_{i},\beta ,\gamma )\\ \;=\{\begin{array}{l} f_{0}(0;z_{i};\gamma )\;\mathrm{if}\;y_{i}=0\\ (1-f_{0}(0;z_{i},\gamma ))\cdot \frac{f_{\mathrm{count}}(y_{i};x_{i},\beta )}{1-f_{\mathrm{count}}(0;x_{i},\beta )}\;\mathrm{if}\;y_{i}>0 \end{array} \end{array}$$
  where β and γ are the corresponding regression coefficients of x in the count model and z in the binomial model, respectively, that can be estimated by maximum likelihood [13,14]. In our study, we focused on the *RR* $=e^{\hat{\beta }}$ estimated, where the $\hat{\beta }$ is estimated from the Hurdle model and the RR estimate is used as the weight of aggregating the effects of each term on the retweet count. The analysis was conducted using the pscl package in R [13,15].
2. Each column (i.e., term) in the DTM dataset was treated as a predictor. Using “Retweet_Count” as the outcome of interest (i.e., $y_{i}$ = Retweet Count of the $i^{th}$ tweet), the univariate model was fit for each term (i.e., $x_{i}\;$= Frequency of the term in the $i^{th}$ tweet) to estimate the relative risk. A term with a higher slope estimate has a stronger association with the number of times a tweet has been retweeted. The *RR* was calculated by taking the exponential of the regression slopes, and an elbow plot was created to display the *RR*s visually.
3. The *AIS* was calculated to measure the overall influence of each tweet, as follows:
  - The DTM dataset was imported and transposed as a TDM. In the TDM, rows referred to terms, and columns referred to documents.
  - The frequency of each term was multiplied by its *RR* to obtain the score for each term. That is, the propensity score was equal to the multiplication of the frequency of a term in a tweet by the *RR* of that term.
  - The summary statistics of the propensity scores of all terms in a corresponding tweet, such as the mean, median, or sum, were calculated. For example, the *AIS*-*mean* score is calculated as the sum of the product of the frequency of a term and the *RR* of that term across all terms in a tweet, divided by the total number of terms in that tweet. Below is a worked example of the calculation of *AIS* scores for a single tweet: 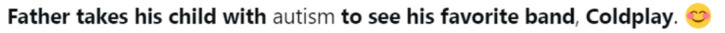

After data preprocessing, the tweet’s text body became “father take his child with to see his favorite band coldplay”. It was transformed to count data in DTM format as follows:**Word****Father****Take****His****Child****with****to****See****Favorite****Band****Coldplay**Frequency in this specific tweet ($n_{i}$)1121111111Frequency in all tweets360104331659231323368349428

Only three words (take, child, and see) in the sample tweet appear in more than 500 tweets. The *RR*s for these are 3.621, 4.312, and 4.529, respectively. We then can calculate the *AIS* based on the mean, median, or sum of *RR*s following Equations (2), (3), and (4), respectively:

(2) $$AIS_{mean}=\frac{\sum_{i=1}^{k}n_{i}RR_{i}}{\sum_{i=1}^{k}n_{i}}$$

(3) $$AIS_{median}=\{\begin{array}{c} RR_{\left(\frac{\sum_{i=1}^{k}n_{i}}{2}+0.5\right)},\;\mathrm{if}\;k\;\mathrm{is}\;\mathrm{odd}\\ \left(RR_{\left(\frac{\sum_{i=1}^{k}n_{i}}{2}\right)}+RR_{\left(\frac{\sum_{i=1}^{k}n_{i}}{2}+1\right)}\right)/2,\;\mathrm{if}\;k\;\mathrm{is}\;\mathrm{even} \end{array}$$

(4) $$AIS_{sum}=\overset{k}{\underset{i=1}{\sum }}n_{i}RR_{i}$$

where $RR_{\left(i\right)}$ indicates the $i^{th}$ order statistics of *RRs* and *k* is the number of unique terms in the corresponding tweet. For example, the *AIS*-*mean* and *AIS*-sum can be obtained using *RR*s and word frequencies following Equations (2) and (4):

$$AIS_{mean}=\frac{3.621\times 1+4.312\times 1+4.529\times 1}{1+1+1}=4.1541;\;$$

$$AIS_{sum}=3.621\times 1+4.312\times 1+4.529\times 1=12.4622.\;$$

For the *AIS*-*median*, we first rank the words by their *RR*s from the smallest to the largest; we place $n_{i}$ entries of *RR* if the word frequency ($n_{i}$) is larger than one.**Term****Child****See****Take**RR × Frequency3.62104.31224.529Place1st 2nd 3rd 

The value at the middle (assuming an odd number of terms, *k*) is the median across the terms, and therefore we obtain the *AIS*-*median* as shown in Equation (3) below:

$$AIS_{median}=RR_{\left(2\right)}=4.3122\;$$

The *AIS* scores were considered as predictors for measuring the influence of terms on the popularity of a tweet on the Twitter platform. Due to zero or extremely small frequencies for certain terms appearing in the entire Twitter data set, *RR*s for those terms were not generated and gave NAs in the dataset. These words provided little information about the association analysis and were excluded from the *AIS* calculations for that tweet.

4 The final step evaluated the combined effect of all terms in a tweet on the popularity of the tweet in terms of retweet counts. For making inferences about the association, the univariate Negative-Binomial or Hurdle model was fitted again using the *AIS* as the predictor, where *X = AIS*. The slope estimate ($\hat{\beta }$) based on Equation (1) of the *AIS* score indicates how strongly the summarized content by *AIS* affected the retweet counts.

#### 2.3.2. Case Study II: Topic Classification by Logistic Regression Model Using ASD against Non-ASD Topics as the Binary Outcome

In this case study, the outcome of interest was whether a tweet belonged to the ASD topic or not. Additional tweets of similar sample size (n = 55,315) to the ASD dataset were randomly selected from the combined dataset of two unrelated non-ASD topics: (1) tweets with the word “influenza” (or “#influenza”) and (2) tweets with the words “violence against women” (or “#violenceagainstwomen”). The data were then merged with the ASD dataset, and an indicator variable was created as 1 = Yes, pertinent to ASD, or 0 = No, pertinent to non-ASD. After data cleaning, a univariate logistic regression model was fitted with each of the 376 terms from the combined dataset. Thus, 376 *OR*s were calculated. The *OR*s were the index used to quantify the effect of classification on the topics. A higher value of the ORs indicated a stronger association between the term and the ASD topic (1 = Yes). A propensity score was then calculated for the individual term by multiplying the *OR* of the term and the frequency with which the term appeared in a single tweet (i.e., *propensity score = OR × frequency*). The propensity score was considered as an overall contributor to the topic. Finally, the propensity scores were summarized as the *AIS* for each tweet. Logistic regression was applied to estimate the AIS, and receiver operating characteristic *(ROC*) curve analysis was then conducted to evaluate the performance of the classification method. In summary, we performed regression analysis as follows:

1. Tweets from the ASD topic and the non-ASD topics, in this example, “influenza” and “violence against women”, served as case and control groups, respectively. Both groups were randomly selected with the same sample size. The new dataset combined the ASD and the non-ASD data with a new variable serving as the outcome of interest, which was an indicator of the tweet’s true classification status (case = 1, pertinent to ASD topic; control = 0, pertinent to non-ASD topics).
  (5) $$\log\left(\frac{\pi }{1-\pi }\right)=\beta_{0}+\beta_{1}X$$
  where π=Pr(Y=1) is the probability of a tweet belonging to topic ASD, and $\beta_{0}$ and $\beta_{1}$ are the coefficient estimates for logistic regression. The logistic regression analysis was performed using the stats package in R (v3.6.0) [10].
2. *OR*s were then calculated using univariate logistic regression (i.e., *Y* = the tweet’s true classification status; *X* = frequency of the term) following a similar process as in steps 1–2 in Case Study I. Here, $\beta_{1}$ obtained from previous step is the coefficient estimated for the *OR* of each term, where $OR=e^{\beta_{1}}$. This *OR* estimate is the weight of each term for calculating the aggregated *OR* score for each tweet. An elbow plot was also created to display the *OR*s visually. The AIS was then calculated for each tweet based on the *OR*s following the same method as in Case Study I, step 3. Specifically, the AIS was calculated inputting *OR*s estimated from Equation (5) instead of using RRs in Equations (2)–(4).
3. The Kruskal-Wallis rank-sum test was conducted to examine the association between the AIS and the outcome of interest. The test evaluated whether the terms of tweets had an impact on the disease topic and provided information about whether the AIS could be used as an indicator to classify tweets between ASD and non-ASD topics. The *ROC* curve and area under the *ROC* curve (*AUC*) were obtained to assess the performance of the classification using the AIS, using the pROC package in R [16].

### 2.4. Model Diagnostics and Evaluation

In Case Study I, the *AIS* of all tweets was applied to fit the final overall count models, and we evaluated the model fit using the rootograms to visualize if the model addressed the overdispersion of the outcome data. In the meantime, the expected zeros were calculated to assist in checking whether the model offered a good enough fit for the excess zeros in the retweet counts. In Case Study II, the *AIS* of all tweets was applied to fit the final overall logistic regression model. The performance of the topic classification using *the AIS* was then evaluated based on the *ROC* analysis.

## 3. Results

### 3.1. Case Study I

After data cleansing, the final dataset used in the study included 55,315 tweets and 135 terms. In the data aggregation, 2949 observations with retweet counts of zero were excluded for the regression analysis using the *AIS* due to the missing values in the AIS. The missing values were produced because some tweets did not include the terms chosen in the analysis. In other words, some terms were initially removed since the overall frequency of the term in the dataset was less than 500 during data cleansing, so missing values were generated in tweets that only included terms appearing less than 500 times in the whole dataset. Thus, the AIS could not be generated as some tweets did not include any key terms. Table 1 provides the results from the two count models that fit with the *AIS*. The results showed that the Hurdle models provided a better fit to the data, with lower Akaike Information Criterion (AIC) scores than the Negative-Binomial models. In addition, the parameter estimates from the Hurdle models were larger than those from the Negative-Binomial models. In the Hurdle analysis, the RRs were about 6.4, 15.3, and 1.5 for the *AIS-mean, AIS-median,* and *AIS-sum,* respectively. This means that, on average, a unit increase in AIS for a tweet would be a 5.4 times greater number of retweets when using the *AIS-mean* as the predictor. Similarly, using the *AIS-median*, we conclude that, on average, a unit increase in AIS for a tweet would have a 14.3 times greater number of retweets. The difference between the results based on *AIS-mean* and *AIS-median* is that *AIS-median* excludes any potential bias from outliers, so the results based on *AIS-median* are generally more reliable than those based on *AIS-mean*. As mentioned above, the analysis using Poisson models was also performed with the same approach for comparison purposes. The Poisson models using the log-linear link function had *RR*s around 1, and AIC values were extremely large for all three *AIS* measures. The Poisson models using the log-log link function had lower AIC values and higher *RR*s than the models using the log-linear link function. The Poisson models were of significantly worse fit compared to the Hurdle and Negative-Binomial models, which consider fitting for over-dispersed data. In summary, among all the models, the Hurdle model, which considers both over-dispersion and excess zeros in the data, using *AIS-median* as the aggregated predictor, was the best fit, with the lowest AIC and the highest RR.

We also repeated the same analysis with the data, this time including hashtag terms. The results showed that the Hurdle model with *AIS-median* gave the best model fit compared to others, which was consistent with the results from models of the dataset without hashtags; however, the models provided a worse fit of the dataset including hashtags than the dataset without hashtags, based on the AIC values. The AIC values from all models of the dataset including hashtags were higher than those from models of the dataset without hashtags.

**Figure 2:**
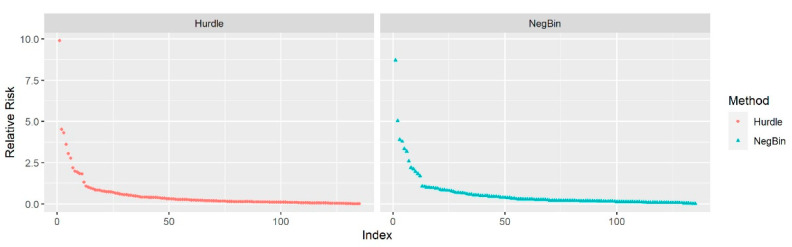
The elbow plot of relative risks of 135 terms from the Hurdle model (**red**) and Negative-Binomial model (**blue**).

**Figure 3:**
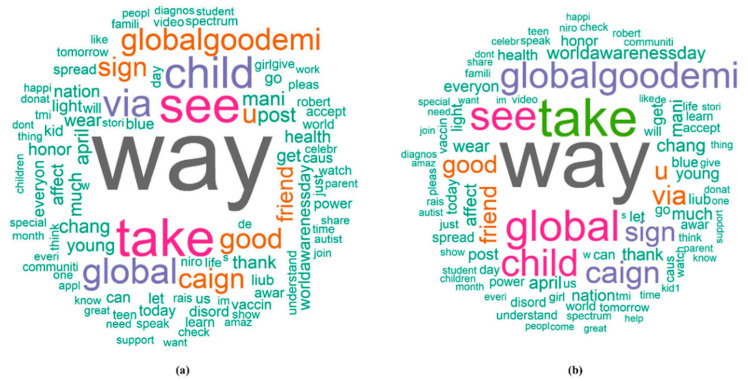
The word clouds of 135 terms (the size of word is determined by *RR*) from the (**a**) Hurdle model and (**b**) Negative-Binomial model.

The elbow plot in Figure 2 provides a visual summary of the *RR*s obtained from univariate analysis using the 135 terms. The elbow plots show that the majority of the terms had an *RR* and *OR* around 1, reflecting minimal effect from these terms if by themselves on the retweet counts. However, the overall effect of multiple terms combined provided more significant and sensible results in evaluating the association between key terms and retweet count. The word-cloud in Figure 3 depicts *RR*s from the Hurdle and Negative-Binomial models, showing the top key words that had a positive effect on retweet count. In addition, we also generated word-clouds of *OR*s from the Hurdle model, which gave the top keywords that had a positive effect on whether a tweet would be retweeted. The size of the text indicated the magnitude of the values of *RR* or *OR*. The larger the text displayed in the word-cloud, the more retweet counts or more likely a tweet containing this word would be retweeted (i.e., retweet count > 0). From the word-clouds, tweets including terms such as “global”, “globalgoodemi”, “doesntendat5”, “worldawarenessday”, and “caign” (short-form of “campaign”) would be more likely to be retweeted, and tweets including terms such as “way”, “child”, “take”, “see”, “global”, “globalgoodemi”, and “caign” would be more likely to have higher retweet counts. Although the word-clouds of the Hurdle count model and Hurdle zero model are different, they are more complementary than contradictory. Therefore, due to consistency between word clouds based on *RR* and *OR*, we presented only the word clouds based on *RR*s (Figure 3).

### 3.2. Case Study II

**Figure 4:**
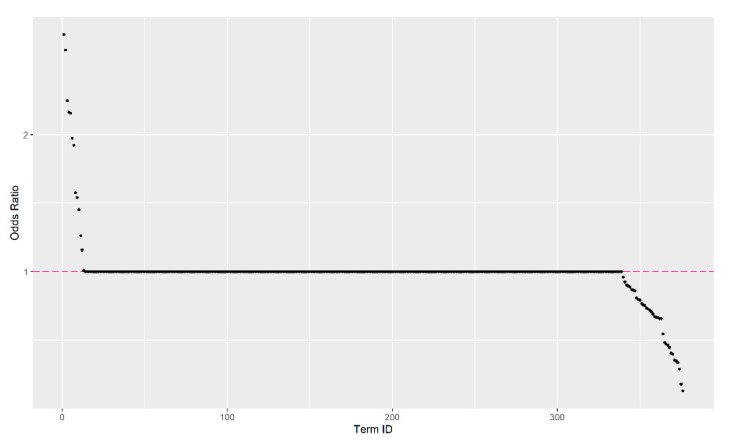
The elbow plot of odds ratios of the significant terms estimated from the univariate logistic regression model. Notice that most terms are not useful for classification, which highlights the advantage of using the *AIS* of keywords (significant terms) for analysis.

In this study, a data set of the same sample size as the ASD tweet data set was randomly selected from tweets with the non-ASD topics “influenza” and “violence against women”. The data were merged with the ASD data and included 110,630 tweets with 376 terms (that were in both ASD and non-ASD groups) as the predictors in the univariate logistic regression model. The logistic regression fitted the probability of the ASD group; therefore, larger *OR* indicates the corresponding term has greater likelihood to appear in a tweet from the ASD group. The *OR*s of all terms were visualized in the elbow plot (Figure 4), with the value of one as the reference line, along with the word-clouds using different reference groups. An *OR* larger than one indicated that the term was more associated with the ASD topic. In contrast, an *OR* less than one showed that the term was more associated with the non-ASD topics. Forty-six of the terms showed a significant association with the ASD topic in the univariate logistic regression. Like Case Study I, we calculated the AIS score for each tweet in terms of mean, median, and sum. The summary statistics of AIS scores are presented in Table 2. Clearly, the sum is not apt for classification, as the *AIS-sum* is higher in the non-ASD group. The reason is that there are more terms with *OR* values significantly less than one (more associated with non-ASD) compared to terms greater than one (with ASD). The sum does not consider the number of terms that were used to summarize a tweet. On the contrary, the median and mean are valid AIS scores for such reasons.

**Figure 5:**
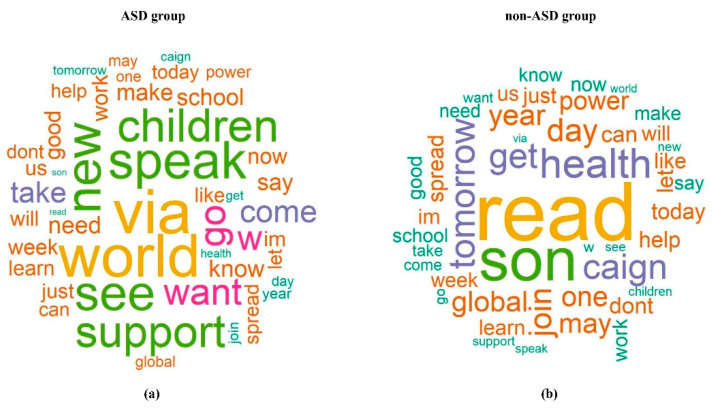
The word clouds of the significant terms from univariate logistic regression in the (**a**) ASD group versus (**b**) non-ASD group. The font size displays the popularity of a particular term in either group: the larger the font size, the larger the values of the odds ratio and hence the greater the presence/popularity of a topic/term.

After model classification, we identified top keywords in each of the ASD and non-ASD groups. The results of topic classification analysis, presented in Figure 5, can be used to assist health practitioners in identifying health issues via social media. Table 2 gives summary results of *AIS* scores from the final logistic models. We can see that the ASD group had on average smaller *OR*s based on *AIS* scores, and they were significantly different from the non-ASD group.

## 4. Model Diagnostic and Evaluation

**Figure 6:**
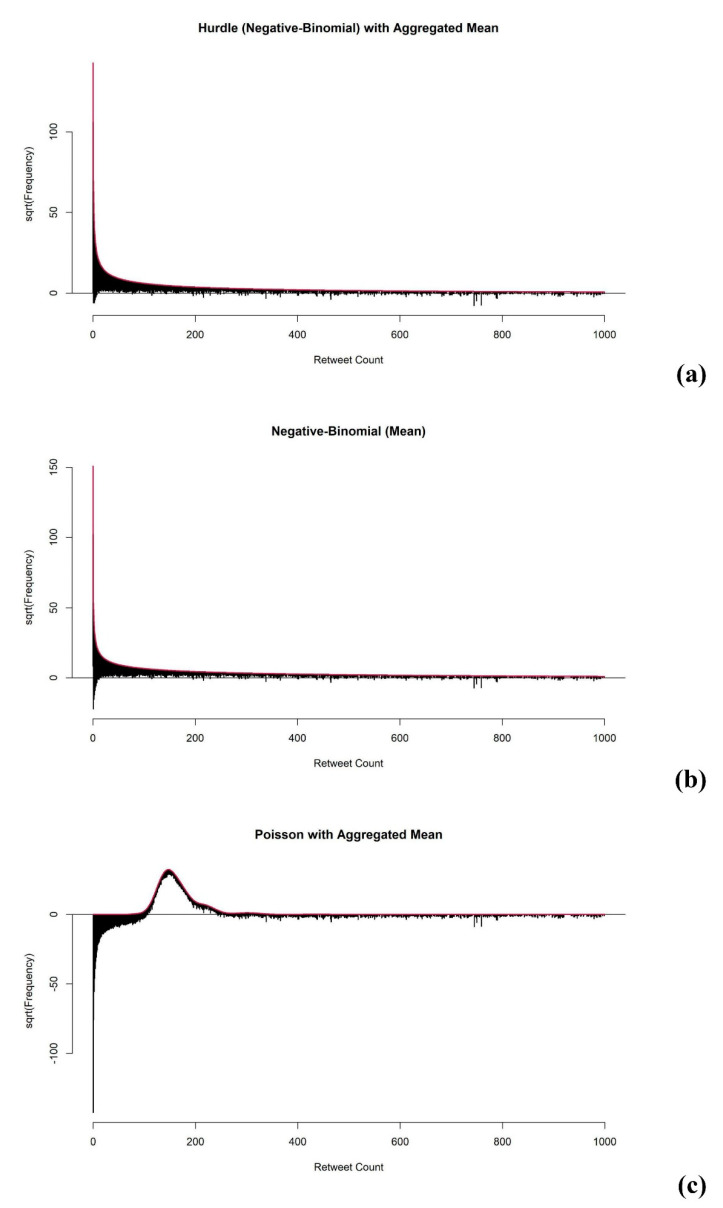
The rootograms from the (**a**) Hurdle model, (**b**) Negative-Binomial model, and (**c**) Poisson model.

The aim of model diagnostic and evaluation for the case I type of association studies is to check whether the model can fit Twitter data with the common problems of over-dispersion and excess zeros. First, to evaluate whether the fitted model addressed the overdispersion of the outcome data well enough, we obtained the rootograms to visualize the overdispersion (see Figure 6) based on the analysis of Kleiber and Zeileis [17]. As mentioned above, the Poisson model has a limitation in that it cannot fit data with over-dispersion well. We provide rootograms of the Poisson model as a reference to compare the model-fitting of the Hurdle and Negative-Binomial models.

From Figure 6, we conclude that, in contrast to the Poisson model, both the Hurdle and Negative-Binomial models addressed over-dispersion well, as most of the observations aligned closely around the horizontal line at 0.

To check the mode fit for excess zeros, the difference between the true number of zeros and the expected number of zeros was obtained. The difference (*D*) of all univariate analysis using Hurdle models was zero, which confirmed that the Hurdle model separated the zeros and non-zeros in the analysis. By the structure of the Hurdle model, it always predicts the same number of zeros from the observed data. Therefore, the Hurdle models using *AIS* gave zero *D*, as expected. On the other hand, the *D* of univariate analysis of each term using the Negative-Binomial model was not zero and had an average of −3154, which indicated that the univariate Negative-Binomial model predicted 3154 fewer zeros on average. The Negative-Binomial model predicted 2452, 2424, and 2355 fewer zeros using *AIS-mean, AIS-median,* and *AIS-sum*, respectively. The estimation based on the Negative-Binomial model improved, as the predicted number of zeros was closer to the true number of zeros in the dataset.

In conclusion, the Hurdle model offered better fitting than the Negative-Binomial model when analyzing text data from Twitter, especially using the *AIS-median* as the predictor.

**Figure 7:**
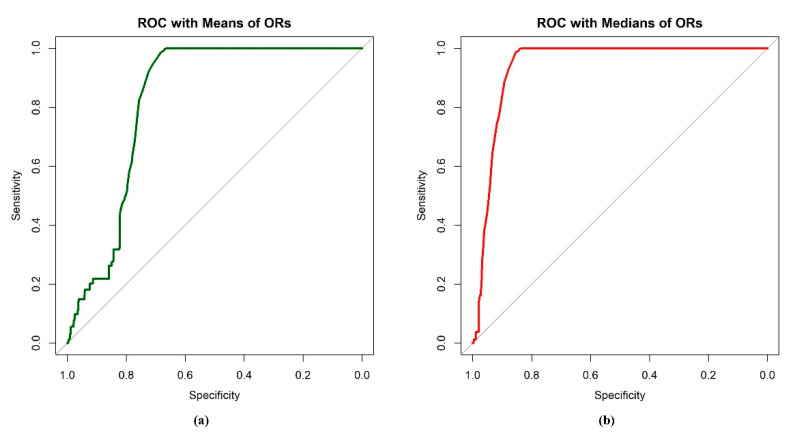
The *ROC* curve for classifying ASD and non-ASD topics based on (**a**) *AIS-mean* and (**b**) *AIS-median*.

For the case II type of classification studies, the *ROC* and the corresponding *AUC* were obtained to assist the evaluation of classification performance based on the *AIS*. The results are summarized in Table 3 and demonstrated in Figure 7. The results indicated that the *AIS-median* had the best performance, with the highest sensitivity and specificity. The *AIS-mean* performed well, yet the specificity was a little lower than the *AIS-median*. The performance of the *AIS-sum* was poor, with invalid *ROC* and *AUC* values, and the sensitivity and specificity were very low compared to the others. To better display the results of the *AIS-sum*, analysis was also conducted to evaluate the performance of classification on the reciprocal of the *AIS-sum*. The *ROC* analysis confirmed that the distributions of *AIS-mean* and *AIS-median* in two different groups were different; also, the two *AIS* scores had the ability to discriminate tweets from the two groups. Moreover, *AIS-mean* and *AIS-median* performed better in discriminating between tweets on ASD and non-ASD topics than *AIS-sum*.

## 5. Discussion and Conclusions

This study aimed to investigate the feasibility of summarizing text messages posted on social media like Twitter through regression models to study the health issues and other general topics of conversation that people are interested in. We proposed new measures based on results from generalized linear models (i.e., Negative-Binomial, Hurdle, and logistic regression models) that are easy to use and generate easily interpreted results, facilitating their use by public health professionals for investigating the associations between terms used in tweets and health issues as well as for classification of topics based on terms from tweets. Additionally, the measure using text-mining data from social media was automatically harvested by computational programs to avoid human mistakes that can occur during manual data entry.

The terms in the word clouds in Figure 5 that frequently presented in the ASD topic were related to children and the World Autism Awareness Day, such as “children”, “world”, ‘’see”, “speak”, and “support”. Terms, such as “health”, “son”, ‘’get’’, ‘’caign’’, and “read” presented more frequently in the non-ASD topics (influenza and women violence campaign). The term “son” was believed to be more associated with the ASD topic, as past ASD studies have shown that boys are more likely to develop ASD than girls [18,19]. Such inconsistency may be because the non-ASD topics selected in this study were influenza and violence against women, which also are issues prevalent among families. It is quite possible that people who have these issues mention their sons more often in social media compared to people who have an ASD issue in the family. In this study, we transformed individual terms from tweet text into continuous data, i.e., the AIS score, using parameter estimates from the generalized linear regression models. At the same time, we were able to combine multiple predictors into a single index that buffered the extreme values among the terms in a tweet. This technique provides clues for future studies to include some variables that are apparently associated with the primary outcome but do not overwhelm/dominate the effects of other variables, i.e., not an influential effect. For example, as we have discussed, the term “son” can be misleading if we classify based on individual terms. Similarly, the word “children” is visibly associated with ASD, and sometimes a non-ASD tweet is misclassified when it contains “children” in the text. In this case, the proposed AIS score based on the presence of other shared terms of a topic will support and contribute to a more accurate and robust classification.

The classification performance of the proposed method based on *AIS* is quite satisfactory. For example, 92.89% of tweets from the ASD group were correctly classified as ASD topics and 87.86% of tweets from the non-ASD control group were correctly classified as non-ASD topics based on AIS-median in our study. In another ASD Twitter study [1] that investigated the common text mining approaches for classifying the ASD tweets and non-ASD tweets from the control group, the highest classification rates among all combinations of methods were 74% for ASD topics and 85% for the control (refer to Table 1 of [1], which used the binary representation of terms and logistic regression for classification). Furthermore, in a study that applied the deep learning approach, Convolutional Neural Network (CNN), for predicting hospitalizations, the *AUC* value of the classifier was 0.83 [20], and in our study, the *AUCs* achieved by *AIS-median* and *AIS-mean* were 0.94 and 0.82. Therefore, these indirect comparisons might suggest the consistent and robust performance of the proposed *AIS* method for classification problems. Nevertheless, since the competing studies used different data sets, we cannot say which method is more accurate and preferred. In summary, this study offers some insights on the feasibility of applications of GLM regression for identifying potential keywords for topics of interest for short messages on social media.

One of the common machine learning techniques for text data is sentiment analysis, which shares a similar idea with our approach in summarizing the text into a continuous score, i.e., the sentiment score, for a sentence, such as a tweet. Some popular supervised models used for sentiment analysis are naïve Bayes [21], K-Nearest Neighbor (K-NN), [22] and random forest [23]. With pre-specified sentiment labels, a model/classifier is trained and then is used to identify the sentiment. The method in the current study is similar to the supervised sentiment analysis in that the use of the univariate RR/OR value is comparable to the pre-specified sentiment label (each positive term counts as + 1, neutral as 0, and negative as—1) for each term: a larger value indicates stronger association between a term and the popularity/topic of tweets. For topic determination and classification, such as case II in our study, unsupervised topic models such as Latent Dirichlet Allocation (LDA) [24] are commonly used by computer scientists. The inputs of LDA are a large group of tweets and the expected number of topics; its output is a list of words ranked based on the probability that a word w belongs to a topic t given a document d, which is calculated by the product of two probabilities: p(word w | document d) * p(word w | topic t). This is somewhat similar to the kernel of the *AIS* calculation in our research, as a product of the word frequency and the corresponding *OR* of the word. The *OR* by univariate regression of each word across all tweets is comparable to *p(word w | topic t)*. The frequency of a word for each tweet is comparable to *p(word w| document d).*

The study has some limitations. First, the performance of the proposed method was not compared against the existing text mining methods for the same data set. Second, the study was restricted to data pertinent to certain topics collected within 1 month. Third, this study only considered the text of tweets for modeling. Other metadata associated with tweets, such as inclusion of web URLs, inclusion of images/videos, self-reported locations, and the number of followers, were believed to be significantly associated with the count outcome of interest, but they were not considered in the current analysis [25,26,27]. Finally, the proposed AIS score categorizes texts using a bag-of-words approach, which pertains to the current data of interest only. Natural Language Processing (NLP) is a fast-advancing field, and recent developments in machine learning techniques have provided many advanced analytical tools for in-depth text data learning. Transformers by deep learning techniques have been the trending NLP methods since 2019. Layers of the neural network are pretrained on unlabeled big data in general to be used for learning the data of interest, and thus transformers are more efficient and robust than other early-stage NLP techniques. For instance, Ormerod et al. [28] adopted the latest NLP method, the transformer language models for comparing the semantic textual similarity (STS), in clinical settings. Arnaud et al. [20] applied the Convolutional Neural Network (CNN) for predicting hospitalizations in the emergency department.

Our future research plan is to formally compare the current approach with some existing text mining methods by analyzing the same data set. In addition, future research can consider other metadata in addition to the text to improve the results of the models and generalize the results to a broader scope. That is, we can extend the current *AIS* score based on tweet text only, i.e., *AIS-text,* to obtain other types of *AIS* scores, such as the sentiment scores [29] of each term, using linguistics-based classifiers [30] and emoticons [31] to obtain *AIS-sent and AIS-pos* using term frequencies from the part of speech (POS) analysis [32], as well as *AIS-url* using terms extracted from the embedded web URLs. In summary, in future research, any spectrum of data can be summarized by the corresponding *AIS* score, and one can investigate a more comprehensive multiple GLM model based on various *AIS* scores derived from different characteristics of tweets, such as in the following model:

$$\begin{array}{l} f\left(\mu_{Y}\right)=\beta_{0}+\beta_{1}AIS_{text}+\beta_{2}AIS_{sent}+\beta_{3}AIS_{pos}+\beta_{4}AIS_{url}\\ \;+\beta_{5}\#images+\beta_{6}\#videos+\beta_{7}\#followers. \end{array}$$

Including the AIS scores on text, sentiment, part of speech, external web links, etc., will greatly reduce the number of predictors in the model and hence reduce the complexity and potentially increase the robustness of the model for prediction of association or classification. Finally, the proposed approach can be readily adapted to other data platforms such as the Electronic Medical Record (EMR) system to perform similar text-mining analysis. Moreover, the proposed method can be used to study communication messages between healthcare professionals and patients. For example, Barracliffe et al. [33] studied communication between healthcare professionals and breast cancer patients to estimate the relationship between patients’ emotions and health outcomes.

In conclusion, this study proposes and explores the feasibility of a novel yet straightforward method by regressing the outcome of interest on the aggregated influence scores for association and/or classification analyses. This method will enable health professionals to analyze and classify tweets or other text documents using GLM regression, which is commonly used for statistical data analysis in public health, and thereby will make digital health research more accessible to public health professionals.

## Figures and Tables

**Table 1:** Summary statistics from the final regression models to evaluate the association of the proposed summary score AIS on retweet frequency.

Model	AIS	Parameter Estimate	Standard Error ^1^	RR	AIC
Negative-Binomial	Mean	0.9340	0.0187	2.5447	325,978
	Median	0.2012	0.0216	1.2229	327,007
	Sum	0.1838	0.0052	1.2018	325,993
Hurdle	Mean	1.8552	0.0527	6.3930	314,887
	Median	2.7288	0.0834	15.3145	314,673
	Sum	0.4304	0.0091	1.5379	313,852

^1^ The standard errors are so small that the confidence intervals are very close to the estimated relative risks. All *p*-values of testing *RR* = 1 are less than 2 × 10^−16^. Therefore, the *AIS* is strongly associated with the retweet frequency, thus offering a good summary measure of the text contents.

**Table 2:** Summary statistics from the final logistic regression models to compare the proposed summary score AIS on the resulting classified groups.

Model	AIS	Mean of AIS		Median of AIS	
		Non-ASD	ASD	Non-ASD	ASD
Logistic	Mean	1.04	1.10	1.00	1.00
	Median	1.02	1.05	1.00	1.00
	Sum	5.15	3.75	4.00	3.00

The standard errors are so small that the confidence intervals are very close to the estimated relative risks. All *p*-values of the Wilcoxon rank sum test of comparing the AIS score among the ASD and non-ASD tweet groups are less than 2 × 10^−16^. Therefore, the two groups are well classified.

**Table 3:** The summary statistics of classification accuracy for AIS scores based on mean, median, and sum.

AIS Scores	AUC	Sensitivity	Specificity	Cut-Off Point
Mean	0.8228	0.9208	0.7246	1.000
Median	0.9396	0.9289	0.8786	1.000
Sum	0.3895	0.5601	0.3767	3.000
Reciprocal of Sum	0.6055	0.7185	0.5082	0.2065

## Data Availability

The data are available upon request. Please email corresponding author at jyin@georgiasouthern.edu.
